# Putrescine Treatment Delayed the Softening of Postharvest Blueberry Fruit by Inhibiting the Expression of Cell Wall Metabolism Key Gene *VcPG1*

**DOI:** 10.3390/plants11101356

**Published:** 2022-05-19

**Authors:** Xiangchong Song, Hongyu Dai, Siyao Wang, Shujuan Ji, Xin Zhou, Jianan Li, Qian Zhou

**Affiliations:** 1 College of Food, Shenyang Agricultural University, Shenyang 110866, China; songxiangchong1020@163.com (X.S.); daihongyu626@stu.syau.edu.cn (H.D.); jsjsyau@syau.edu.cn (S.J.); zhou_xin870828@syau.edu.cn (X.Z.); lijianan0312@163.com (J.L.); 2 School of Public Health, Shenyang Medical College, Shenyang 110034, China; wangsiyao20221@163.com

**Keywords:** blueberry, putrescine, softening, cell wall metabolism

## Abstract

The postharvest shelf life of blueberries is very short at room temperature owing to softening, which reduces their edible value. Putrescine (Put) plays an important role in maintaining the firmness and prolonging the storage time of fruits. Therefore, we investigated the relationship between Put and the cell wall metabolism and their roles in the postharvest softening of blueberry. Harvested blueberry fruit was immersed in 1 mM Put aqueous solution for 10 min. After treatment, the blueberries were stored at 20 ± 0.5 °C and 80% relative humidity for 10 days. The results show that Put delayed the softening of the blueberries. Compared to the control, the blueberry fruit treated with Put showed higher levels of firmness and protopectin. Moreover, the activity and expression levels of the cell wall metabolism enzymes were markedly inhibited by the Put treatment, including polygalacturonase (PG), β−galactosylase (β−Gal), and β−glucosidase (β−Glu). The Put treatment promoted the expression of the Put synthesis gene *VcODC* and inhibited the expression of the Put metabolism gene *VcSPDS*. Further tests showed that the fruit firmness decreased significantly after the overexpression of *VcPG1*, which verified that *VcPG1* is a key gene for fruit softening. The key transcription factors of fruit softening were preliminarily predicted and the expressions were analyzed, laying a foundation for the subsequent study of transcriptional regulation. These results indicate that Put delays the softening of postharvest blueberry by restraining the cell wall metabolism and maintaining the fruit firmness.

## 1. Introduction

Blueberry (*Vaccinium* spp.) is a popular fruit that is rich in vitamins, minerals, proteins, and other elements [1,2]. The unique purple blueberry peel is rich in anthocyanins. Moreover, blueberry fruit has the functions of resisting oxidation, lowering blood lipid, improving eyesight, resisting malignant cell proliferation, inhibiting cancer cell growth, and regulating metabolism [3,4,5]. During the storage of blueberries, their sensory quality declines and they are affected by attacks of mildew, which will eventually shorten the shelf life of blueberries. The color, texture, flavor, nutrients, active oxygen metabolism, and antioxidant activity of the fruit will also change accordingly [6]. The poor storability of blueberry severely restricts its commercial value and economic development, which undoubtedly brings huge losses.

Softening is a sign of fruit ripening, which is a complicated process. However, over−softening after harvest will cause economic loss [7,8]. Softening can affect the appearance, texture, and flavor of fruit [9]. Softening may be related to changes in the cell wall structure. Pectin, which is a kind of polysaccharide, is an important component of fruit cell walls. Pectin is found mostly in the cell wall and is abundant in citrus, grapefruit, and lemon peel. At the early stage of shelf life, pectin exists in the form of protopectin (PP). With the ripening and softening of the fruit, the PP is gradually transformed into water−soluble pectin (WSP); the degradation of pectin during fruit ripening is the main reason for fruit softening [10]. The process of cell wall catabolism requires the synergy of pectin, hemicellulose, cellulose, and cell wall metabolic enzymes [11]. Cell wall metabolic enzymes play a crucial role in fruit softening and include polygalacturonase (PG), pectinesterase (PE), cellulase (Cx), and β−galactosidase [12]. Water loss is also an important reason for fruit softening [13]. In recent years, cell wall metabolic enzymes have been extensively studied in the softening process of apples, tomatoes, pears, and litchi [14,15,16,17]. Based on transcriptome technology, many key genes related to softening in cell wall metabolism were identified in a study of fruit postharvest softening. After the transient expression of PavXTH14, PavXTH15, and PavPG38 in cherry fruit, the firmness of the fruit was significantly reduced, and the contents of hemicellulose and pectin were also changed [18]. Therefore, it is important to identify the key genes of cell wall metabolism for the study of fruit softening.

**Figure 1:**
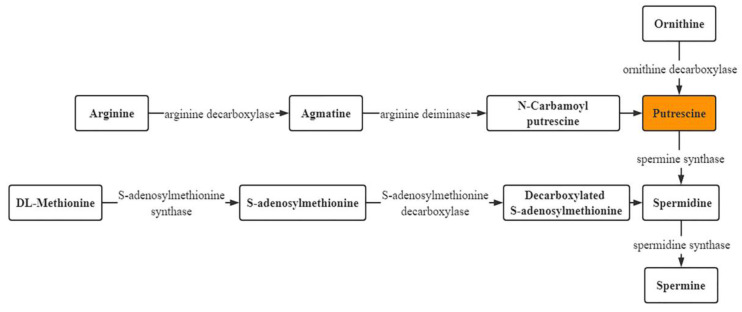
Anabolic pathways of polyamines.

Polyamines (PAs) are ubiquitous nitrogenous bases found in all living organisms and can regulate their growth and development and delay senescence [19]. In recent years, PAs have mostly been used in the storage and preservation of fruits and vegetables, including peach, grape, mango, and zucchini [20,21,22,23]. PAs mainly include putrescine (Put), spermidine (Spd), spermine (Spm), and cadaverine (Cad). As shown in Figure 1 [24], PAs are synthesized in plants in two main ways: (1) Arginine decarboxylase catalyzes arginine to generate agmatine, finally forming Put. (2) Ornithine decarboxylase (ODC) catalyzes ornithine to generate Put. Spermidine synthase (SPDS) catalyzes Put to form Spd. ODC and SPDS play important roles in the metabolism of PAs.

Put has been used in the research on the postharvest softening of fruits and vegetables in recent years, which has a certain effect on the activity of the cell wall metabolic enzymes of fruits. A Put treatment in pepper significantly reduced the activities of Cx and pectin methylesterase (PE) [25]. A Put treatment effectively maintained the firmness of pear and inhibited the degradation of starch and titratable acid and the activity of PE and Cx [26]. A Put treatment in mango inhibited ethylene production and cell wall metabolic enzyme activity and improved antioxidant enzyme activity during fruit ripening, thereby maintaining better fruit quality [27]. To our knowledge, no information is accessible regarding the role of Put in regulating cell wall metabolic enzyme activity and the fruit softening of blueberries after harvest, and therefore, it deserves further investigation. Therefore, this study explored the possible mechanism of Put treatment in the softening of blueberries from the perspective of the fruit cell wall metabolism, exploring the mechanism changes in fruits and providing a new direction for the subsequent preservation development of blueberry.

## 2. Results

### 2.1. Effect of Put Treatment on Decay Incidence and Firmness of Blueberry Fruit during Softening

**Figure 2:**
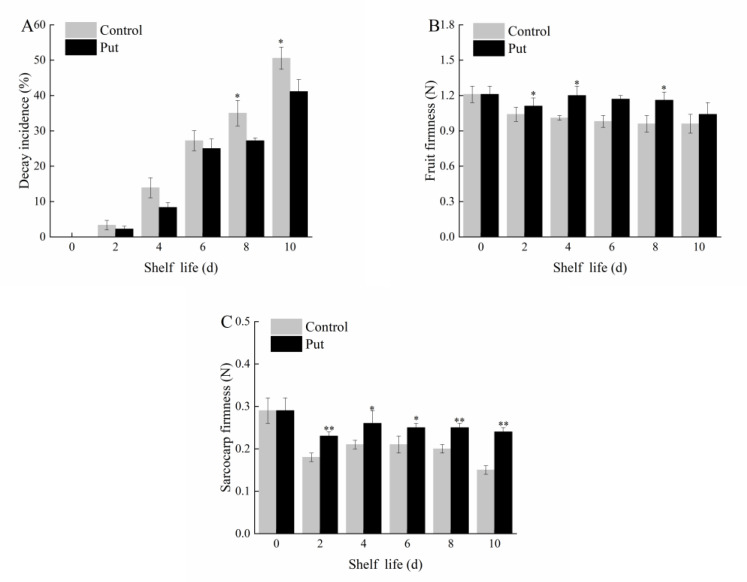
Changes in decay incidence (**A**), fruit firmness (**B**), and sarcocarp firmness (**C**) of postharvest blueberry fruit at 20 °C. Mean ± SE of three replicate experiments are shown. Asterisks indicate significant differences between control and Put−treated fruit (* *p* < 0.05, ** *p* < 0.01). The control was soaked in distilled water for 10 min, the same below.

As can be seen in Figure 2A, the fruit decay incidence of the control and Put treatment groups increased to different degrees. At the end of the shelf life, there was a significant difference between the Put−treated and control groups (*p* < 0.05). The decay incidence in the control fruits exceeded 50% on day 10 of the shelf life, while that in Put−treated fruit was only 41.11%, indicating that the Put treatment reduced the loss by about 9%.

Firmness is not only an important index of the fruit ripening standard and fruit quality but also an intuitive indicator of fruit softening. The firmness of both groups shows a decreasing trend during the shelf life period in Figure 2B,C. After the Put treatment, the firmness of the blueberries on days 2, 4, and 8 was significantly higher than that of the control in Figure 2B (*p* < 0.05), decreasing from 1.21 N to 1.04 N. The fruit firmness of the control decreased from 1.21 N to 0.96 N. After the Put treatment, the sarcocarp firmness decreased slowly and was significantly higher than that of the control in the first 10 days Figure 2C (*p* < 0.05). The sarcocarp firmness was 0.29 N on day 0. The sarcocarp firmness of Put treatment decreased to 0.24 N on the 10th day, and that of the control group decreased to 0.15 N. It can be seen from this result that the Put treatment effectively delayed the decline of the fruit firmness and thus prolonged the edible life of the blueberries.

### 2.2. Effect of Put Treatment on Soluble Solid Content of Blueberry Fruit during Softening

**Figure 3:**
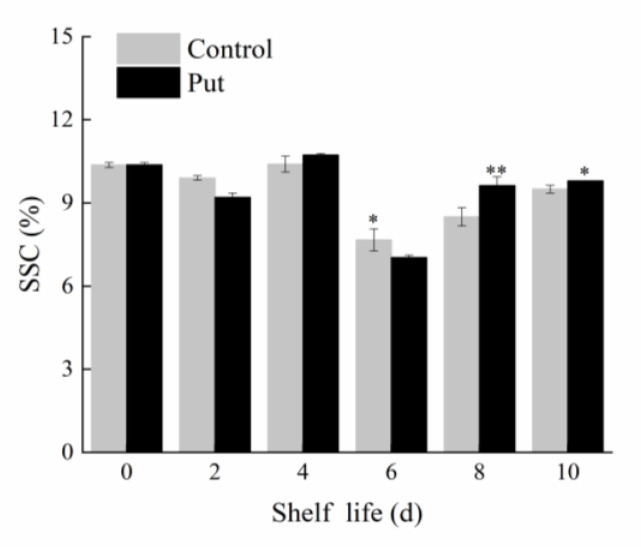
Changes in SSC of postharvest blueberry fruit at 20 °C. Mean ± SE of three replicate experiments are shown. Asterisks indicate significant differences between control and Put−treated fruit (* *p* < 0.05, ** *p* < 0.01).

In general, the higher the soluble solid content (SSC), the higher the sugar content of the fruit. At the beginning of the fruit storage, the SSC was 10.37% (Figure 3). The SSC at the end of the shelf life was lower than that at the beginning of storage, which may have been due to the ripening of the fruit, causing the SSC to decrease; the Put treatment slowed down this process. The SSC of the Put treatment group was significantly higher than that of the control on days 8 and 10 (*p* < 0.05).

### 2.3. Effect of Put Treatment on PP and WSP Content of Blueberry Fruit during Softening

Pectin is found in the cell walls of fruit between the cell layer and the primary cell wall, and it participates in fruit ripening and softening [28].

**Figure 4:**
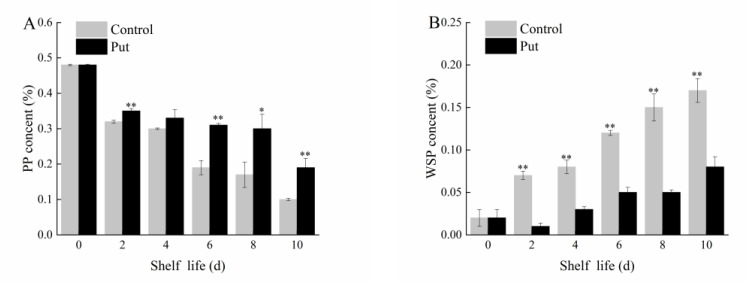
Changes in PP (**A**) and WSP (**B**) of postharvest blueberry fruit at 20 °C. Mean ± SE of three replicate experiments are shown. Asterisks indicate significant differences between control and Put−treated fruit (* *p* < 0.05, ** *p* < 0.01).

In the early stage of the shelf life, the PP content was 0.48% (Figure 4A). In the late stage of the shelf life, the PP content in the control was reduced to 0.10%, and in the Put−treated group, it was reduced to 0.19%. The PP content of the treatment group was significantly higher than that in the control group on days 2, 6, 8, and 10 (*p* < 0.05). The WSP content showed an upward trend during the shelf life. The WSP content was 0.02% in the early stage of the shelf life, and increased to 0.17% in the control and 0.08% in the Put−treated fruit at the end of the shelf life (Figure 4B). The WSP content in the control was significantly higher (2.13 times) than that in the Put−treated fruit (*p* < 0.01). The results show that the Put treatment inhibited the degradation of PP and maintained the fruit firmness.

### 2.4. Effects of Put Treatment on PG, β−Gal, and β−Glu Activities and Related Gene Expression Levels in Blueberry Fruit during Softening

Cell wall metabolic enzymes can catalyze the degradation of PP and reduce fruit firmness, so the activities of related cell wall metabolic enzymes and gene expressions were determined. Three enzymes related to cell wall degradation were analyzed.

**Figure 5:**
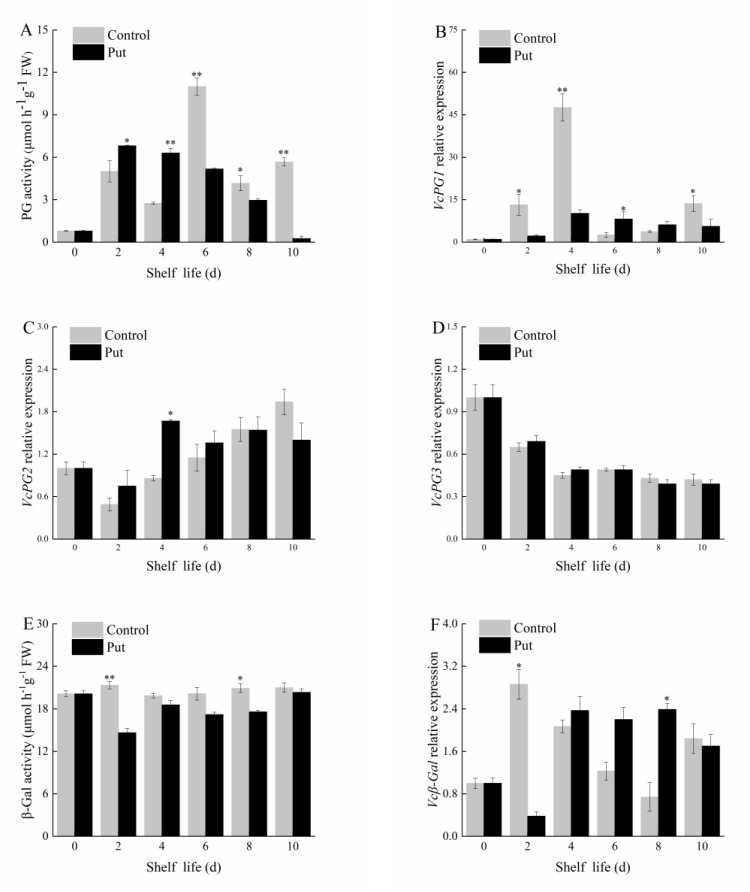

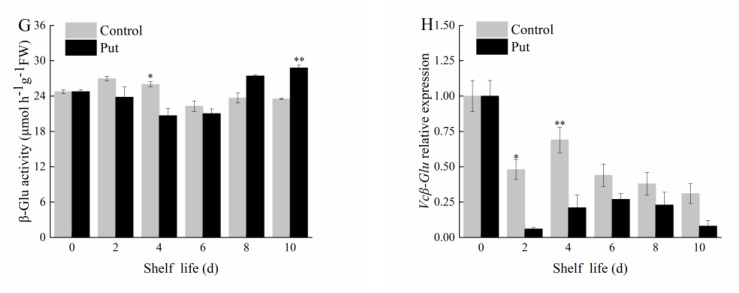
Changes in PG (**A**), β−Gal (**E**), and β−Glu (**G**) activity and *VcPG1* (**B**), *VcPG2* (**C**), *VcPG3* (**D**), *Vcβ−Gal* (**F**), and *Vcβ−Glu* (**H**) relative expression of postharvest blueberry fruit at 20 °C. Mean ± SE of three replicate experiments are shown. Asterisks indicate significant differences between control and Put−treated fruit (* *p* < 0.05, ** *p* < 0.01).

The PG activity in the Put−treated fruit reached a maximum of 6.81 μmol h^−1^g^−^^1^ FW on day 2, and then decreased. The PG activity in the Put−treated fruit was significantly higher than that in the control before day 4 (*p* < 0.05) (Figure 5A). With the extension of the shelf life, the PG activity in the Put−treated fruit was significantly lower than that in the control after day 6 (*p* < 0.05), and the PG activity peak of the control was 11 μmol h^−^^1^g^−^^1^ FW, indicating that the Put−treated fruit significantly inhibited the PG activity and decreased the PG activity peak. The relative expression level of the *VcPG1* gene in the control reached a maximum value of 47.59 on day 4, as shown in Figure 5B, which was significantly higher than that in the Put−treated fruit (*p* < 0.05). The Put treatment reached a maximum value of 10.17 on day 4, and then decreased slowly. The expression of the *VcPG1* gene was significantly inhibited in the Put−treated blueberries on days 2, 4, and 10 (*p* < 0.05). As shown in Figure 5C,D, the Put treatment did not significantly inhibit the expression of the *VcPG2* and *VcPG3* genes.

The β−Gal activity in the Put−treated fruit was always lower than that in the control during the shelf life period, and the change was relatively stable, indicating that the β−Gal activity in the Put−treated fruit was inhibited (Figure 5E). The β−Gal activity of the fruit was 20.10 μmol h^−1^g^−1^ FW in the early storage period. The β−Gal activity in the Put−treated fruit was 20.30 μmol h^−1^g^−1^ FW at the end of the shelf life, slightly lower than that in the control. The relative expression level of the *Vcβ−Gal* gene in the Put−treated fruit was higher than that in the control during days 4 to 8 of the shelf life period, as shown in Figure 5F; in the Put−treated fruit, it was significantly inhibited on day 2 (*p* < 0.05) and changed steadily after day 4. The Put treatment inhibited the *Vcβ−Gal* peak, which reduced the β−Gal activity.

The β−Glu activity in the Put−treated fruit decreased first and then increased; it was 24.76 μmol h^−^^1^g^−^^1^ FW at the beginning of the shelf life and 28.79 μmol h^−^^1^g^−^^1^ FW at the end of the shelf life. It can be seen from Figure 5G that the β−Glu activity in the Put−treated fruit was inhibited before day 6. In general, the relative expression of the *Vcβ−Glu* gene decreased during the normal−temperature storage, as shown in Figure 5H. It was always lower in the Put treatment than in the control, and the inhibition effect was significant on days 2 and 4 (*p* < 0.05).

### 2.5. Effect of Put Treatment on the Expression of Put Anabolism−Related Genes in Blueberry Fruit during Softening

**Figure 6:**
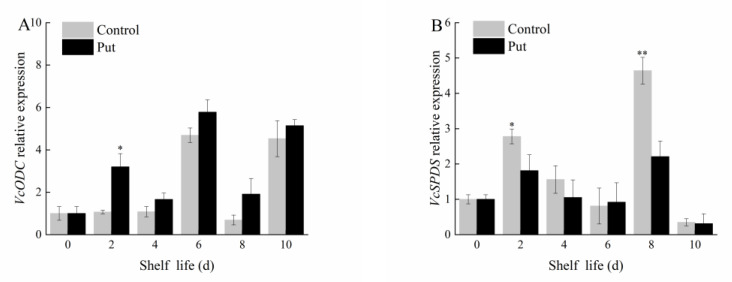
Changes in *VcODC* (**A**) and *VcSPDS* (**B**) relative expression of postharvest blueberry fruit at 20 °C. Mean ± SE of three replicate experiments are shown. Asterisks indicate significant differences between control and Put−treated fruit (* *p* < 0.05, ** *p* < 0.01).

As can be seen in Figure 6A, the expression of *VcODC* in the Put treatment during the shelf life was higher than that of the control group. Therefore, the Put treatment upregulated *VcODC* expression. As can be seen in Figure 6B, after the Put treatment, the relative expression of the *VcSPDS* gene was significantly inhibited on days 2 and 8, and it was still higher in the control than in the Put treatment at the end of the shelf life. Therefore, it can be seen that the Put metabolism was inhibited after treatment. These results suggest that the exogenous Put treatment regulated the endogenous Put anabolism mechanism, affecting the softening process of the blueberries.

### 2.6. Cloning and Analysis of Coding Region VcPG1 Gene

**Figure 7:**
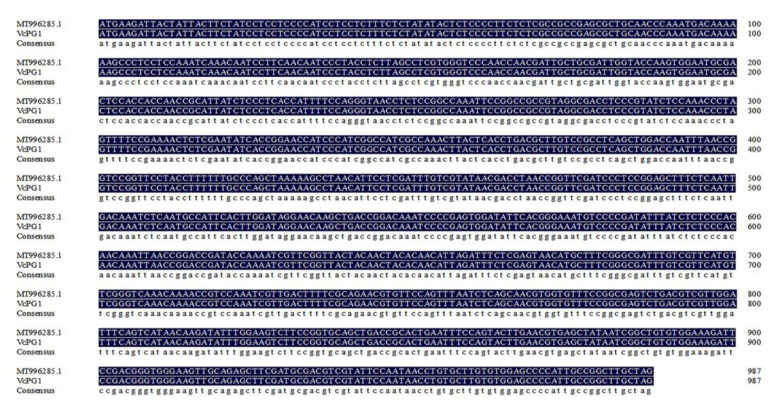
Sequencing result of *VcPG1* full−length coding region. Compared with the mRNA of *Vaccinium corymbosum* polygalacturonase mRNA (MT996285.1) in NCBI, the similarity was 100%.

As shown in Figure 7, the total length of the coding region (CDS) of the *VcPG1* gene by DNAMAN software is 987 bp. NCBI online comparison showed that it had high homology with *Vaccinium corymbosum* polygalacturonase mRNA (MT996285.1), so it could be identified as the coding region sequence of the *VcPG1* gene.

### 2.7. Changes in Morphology and Firmness of Blueberry Fruit Overexpressing VcPG1

**Figure 8:**
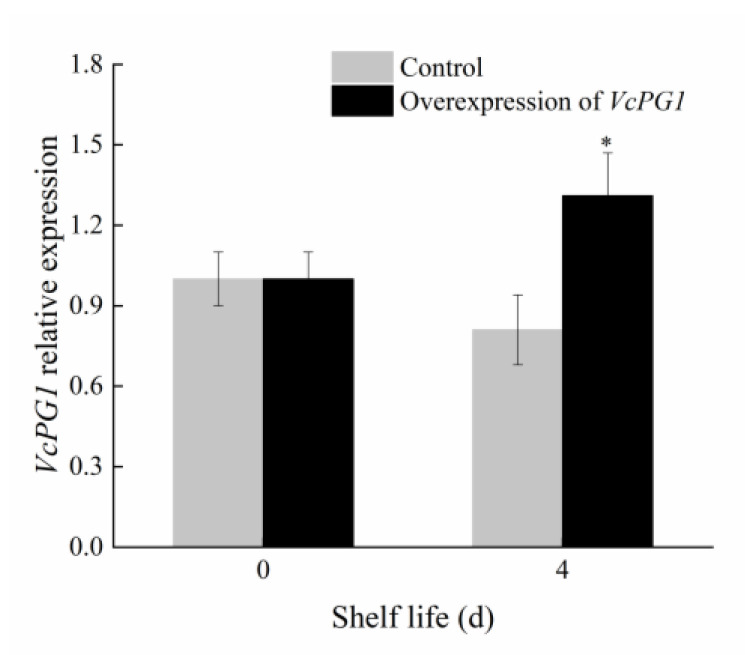
Changes in *VcPG1* expression in *VcPG1*−OE blueberries. The control fruit was injected with empty vector Agrobacterium tumefaciens. Asterisks indicate significant differences between control and overexpressing *VcPG1* treatment fruit (* *p* < 0.05).

**Figure 9:**
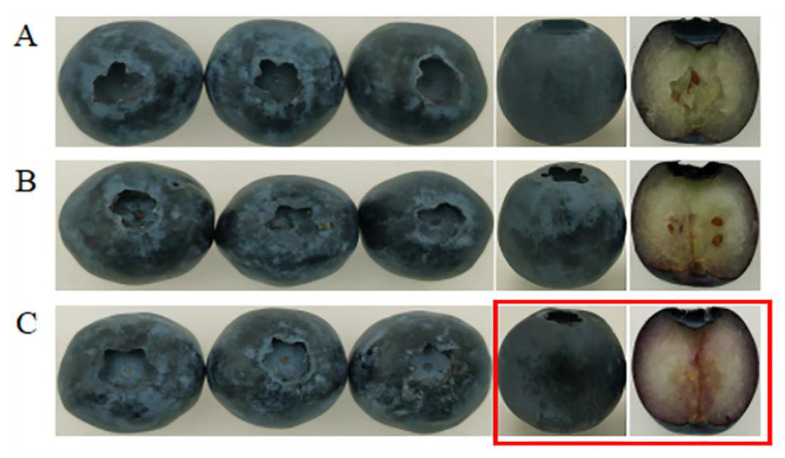
Effects of overexpression of *VcPG1* on the morphology of blueberries at 20 °C. (**A**) Fresh blueberries (0 d). (**B**) Empty vector (20 °C, 6 d). (**C**) Overexpression of *VcPG1* (20 °C, 6 d). The red frame indicates that after the overexpression of *VcPG1*, the softening symptoms were enhanced.

In order to verify that *VcPG1* is the key gene of blueberry fruit softening, a transient transfection experiment was carried out on the fruits. As can be seen from Figure 8, the expression of the *VcPG1* gene in the *VcPG1*−OE fruit was significantly higher than that in the control on day 4 (*p* < 0.05). As can be seen from Figure 9, the comparison shows that the fruits were full on the day of harvest, the outside of the pericarp had a thin white wax layer, and the interior of the fruit was fresh green. On day 6, part of the wax layer fell off and shrunk after the empty vector injection, and the color of the inner pulp became lighter, showing water stains. Compared to the control fruit, the wax layer of the *VcPG1*−overexpressed fruit fell off significantly, the part of the inner fruit was reddish−brown, and the softening symptoms were enhanced.

**Figure 10:**
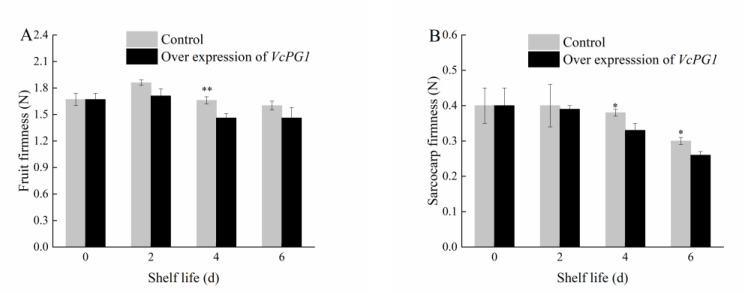
Changes in fruit firmness (**A**), and sarcocarp firmness (**B**) with overexpression of *VcPG1* in blueberries at 20 °C. Mean ± SE of three replicate experiments are shown. Asterisks indicate significant differences between control and treated fruit (* *p* < 0.05, ** *p* < 0.01).

As can be seen in Figure 10, the firmness of the *VcPG1*−overexpressed fruit was always lower than that of the control fruit. The firmness of the control fruit was extremely significantly higher than that of the treated fruit on day 4 (*p* < 0.01), and the sarcocarp firmness of the control was significantly higher than that of the treated fruit on days 4 and 6 (*p* < 0.05). The results show that the *VcPG1* gene was involved in regulating the softening process of the blueberry fruit and promoting a decrease in the fruit firmness.

### 2.8. Prediction of Related Transcription Factor Loci

**Figure 11:**
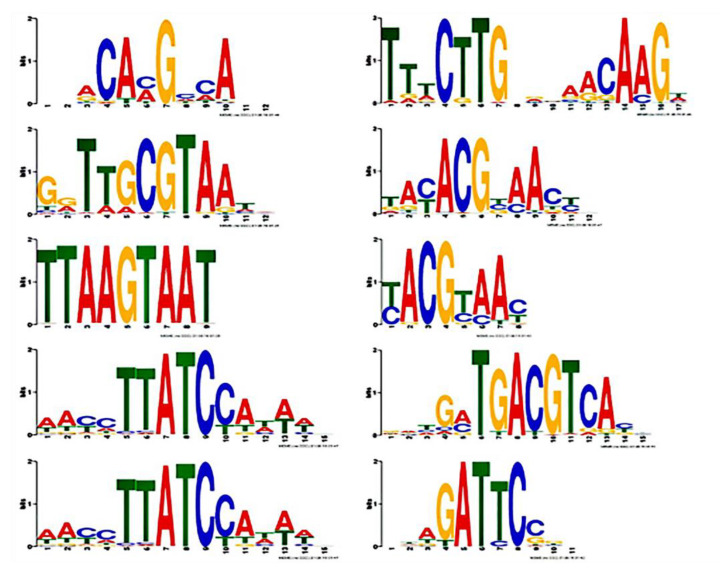
Prediction of possible cis−acting elements bound by key transcription factors VcNAC, VcMYB, VcWRKY, VcbZIP, VcPHL, and other families.

The possible binding base sequences of transcription factors VcNAC, VcMYB, VcWRKY, VcbZIP, VcPHL families were predicted, which laid a foundation for the study of transcriptional regulation in the future. It can be seen in Figure 11 that there are base sequences that predict possible bindings: ACAAG, TTTCTT, CGTAA, TACGT, TTAAG, TACGT, TTTCTTATCCAAA, GCTGAC, AGTCAA, ATCCC.

### 2.9. Analysis of the Expression of Predictive Transcription Factors

**Figure 12:**
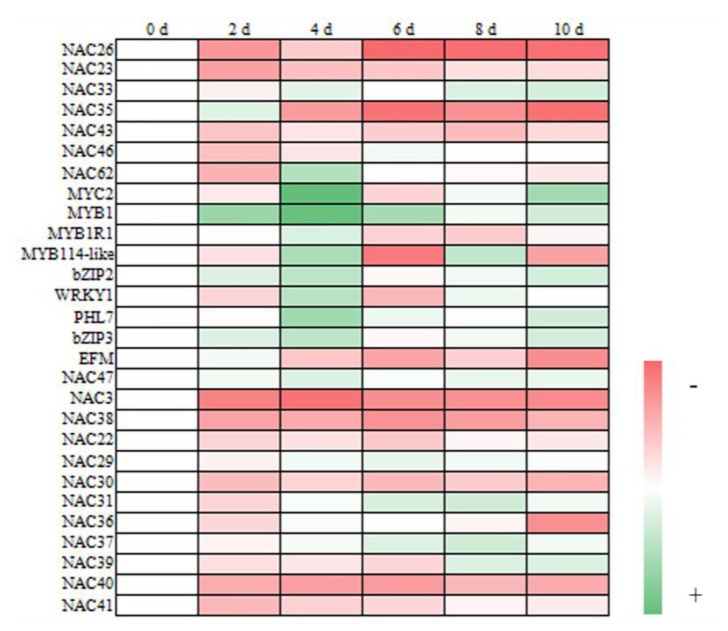
Heatmap analysis of the expression of 28 transcription factors in blueberry fruit.

By analyzing the transcriptome sequencing results, we screened out the transcription factors that may regulate blueberry fruit softening. The primers were designed to determine the expression levels, and the results are shown in Figure 12.

**Figure 13:**
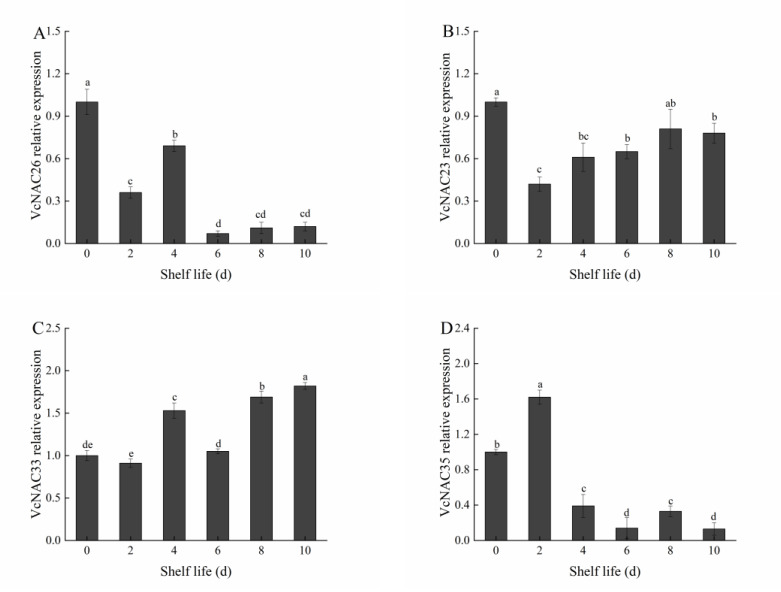

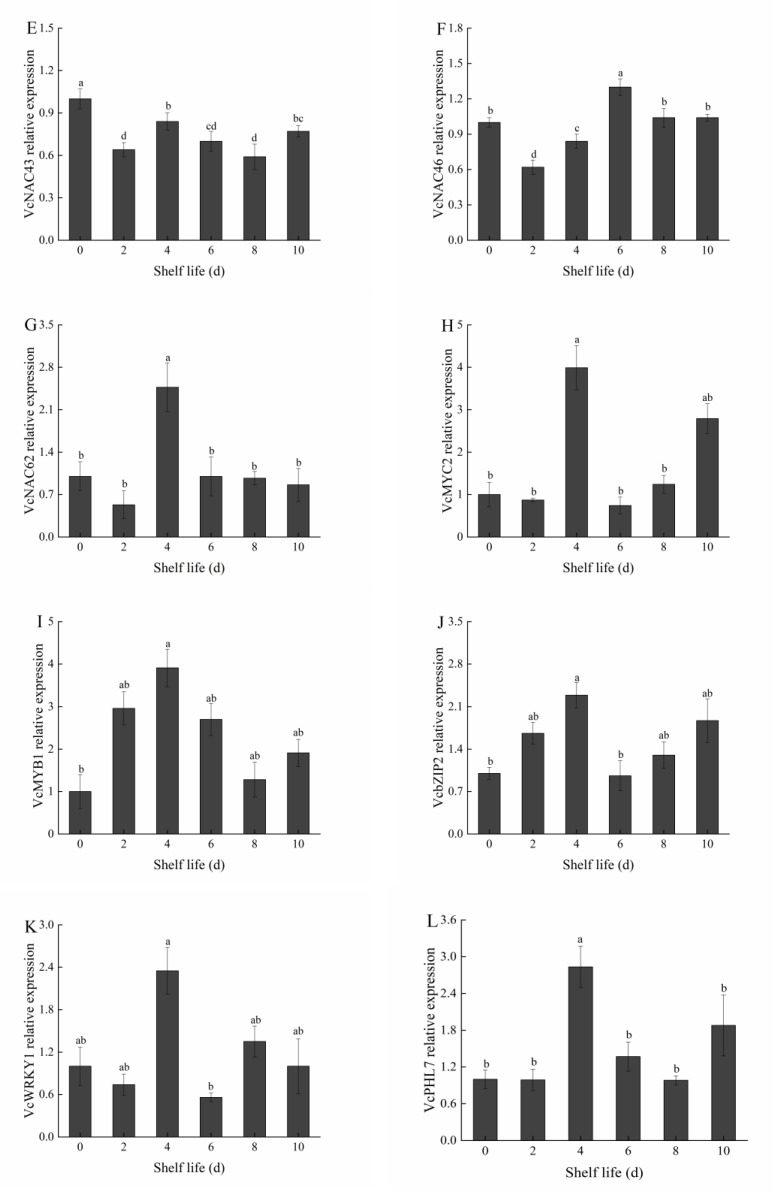
Predicted changes in VcNAC26 (**A**), VcNAC23 (**B**), VcNAC33 (**C**), VcNAC35 (**D**), VcNAC43 (**E**), VcNAC46 (**F**), VcNAC62 **(G**), VcMYC2 (**H**), VcWRKY1 **(I**), VcMYB1 (**J**), VcbZIP2 (**K**) and VcPHL7 (**L**) relative expressions in blueberry fruit after harvest at 20 °C. Mean ± SE of three replicate experiments are shown. Different letters represent significant differences (*p* < 0.05).

By analyzing these 28 transcription factors, we obtained 12 transcription factors with significant differences, as shown in Figure 13.

As shown in Figure 13, the expression of VcNAC26 decreased first, then increased, and then decreased again, and the expression of VcNAC23 decreased first and then increased, and then both tended to be stable. At the end of the shelf life, both of them were significantly lower than on the day of fruit picking (*p* < 0.05). The expression of VcNAC33 changed continuously during the first 6 days and reached a high level after 8 days. The expression of VcNAC35 reached a maximum on day 2, tended to be stable after 4 days, and was much lower than that in the early stage of storage. The change in the expression of VcNAC43 was wavy, and it was at its maximum on the day of fruit picking. The expression of VcNAC46 decreased first, then increased, and then decreased again, and the peak appeared on day 6 of the shelf life. The expression of VcNAC62 decreased first, then increased, and then decreased again. It was at its maximum on day 4, and tended to be stable after 6 days. Among the seven members of the VcNAC family measured in the experiment, the expressions of VcNAC26, VcNAC46, and VcNAC62 tended to be stable at the end of storage and were lower than on the day of fruit picking. The expression of VcMYC2 increased first, then decreased, and finally increased. It reached its maximum on day 4 and was much higher than that on the day of fruit picking. The expression of VcMYB1 increased in the first 4 days and fluctuated up and down after 6 days of storage. The expression of VcbZIP2 increased first, then decreased, and then increased again, reaching its maximum on day 4. The expression of VcWRKY1 was wavy, reaching its maximum on day 4, and there was no significant change between day 10 and the day of fruit picking (*p* < 0.05). The expression of VcPHL7 reached its maximum on day 4, which was significantly higher than that in other storage periods (*p* < 0.05).

## 3. Discussion

The softening of fruit texture is the main process of fruit ripening. Fruit softening can be caused by a decrease in fruit firmness, pathogen infection, decay and deterioration, and changes in the cell wall structure and composition [29]. Banana, persimmon, strawberry, blueberry, and other fruits are easy to soften after harvest [30,31,32,33]. Blueberries have a thin peel and abundant juice and are vulnerable to mechanical damage due, which eventually leads to microbial growth and nutrition and water loss, so the postharvest shelf life of fresh blueberries is very short [34]. In order to prolong the edible period of blueberry fruit, the fruit was soaked with 1 mM Put to explore the physiological changes.

Firmness is an important index of the fruit ripening standard and fruit quality, which affect the shelf life of fruits. In a study of kiwifruit, a 1 mM Put treatment retained the fruit firmness, reduced the fruit respiration, delayed the ethylene production rate, and maintained the overall quality attributes [35]. In this study, a 1 mM Put treatment reduced the decay incidence by approximately 9%, reducing economic losses. The decay incidence of the control fruit on day 8 was significantly higher than that of the treated fruit (*p* < 0.05), and the edibility was reduced. With the extension of the shelf life, the firmness of the blueberry fruit decreased continuously, but the treatment of Put was effective compared to the control. The Put treatment inhibited the decrease in the fruit firmness, and apparently delayed the fruit softening. In general, during fruit ripening, WSP increases, and the contents of PP, hemicellulose, and cellulose decrease [36]. Pectin is an important component of cell walls. The methylation of pectin decreases during fruit storage, the PP transforms into WSP, and the primary wall disintegrates, resulting in a decrease in fruit firmness [37]. Pectin may depolymerize during early to middle ripening, but this change is usually most pronounced during late ripening [38]. After being treated with Put, the firmness and PP contents of blueberries decreased slowly. Cell wall metabolism is closely related to cell wall metabolic enzymes, including PG, PE, Cx, β−Glu, β−Gal et al. [39,40]. During softening, pectin depolymerization is related to cell wall metabolic enzymes. Cell wall metabolic enzyme genes regulate the activities of related enzymes and then catalyze the degradation of PP, resulting in a decrease in the fruit firmness. PG and PE are two main enzymes acting on the pectin part of the cell wall. PE catalyzes pectin demethylation, making the pectin wall easy to be further degraded by PG [41]. In the control, the activity of the PG reached a peak on day 6 of the shelf life, indicating that the PG may have played a greater role in the fruit softening on day 6. After the blueberries were treated with Put, the activity of the PG and the relative expression of the *VcPG1* genes were significantly inhibited, and both of them continued to decline after day 4. However, the Put treatment could not inhibit *VcPG2* and *VcPG3*. Therefore, we speculate that the application of exogenous Put regulated the expression of *VcPG1*, greatly reduced the peak PG activity, and delayed the cell wall degradation, maintaining the firmness of the fruit. After the overexpression of *VcPG1* in the blueberry fruit, the wax layer on the fruit surface fell off significantly, and the firmness significantly decreased compared to the control. Therefore, it can be determined that *VcPG1* is a key gene in reducing the firmness of blueberry fruit. β−Gal can change the cell wall structure and soften fruit. The β−Gal activity of postharvest fruit was inhibited by a MeJA treatment [42]. During the shelf life period of blueberries, the activity of β−Gal in the Put−treated group was always lower than that of the control, indicating that Put inhibited the activity of β−Gal in blueberries. β−Glu is one of the important components of the Cx system, which mainly acts on the non−reducing β−D−glycosidic bond and releases glucose [43]. The results show that β−Glu activity was inhibited within the first 6 days of the Put treatment. Another study also showed that the Put treatment of ‘*Angelino*’ plum before storage can inhibit ethylene biosynthesis and reduce the activities of softening enzymes, such as PE and endo−polygalacturonase [44]. Wang found that ethylene can accelerate blueberry softening and promote the degradation of pectin and the expression of pectinesterase and polygalacturonase [45]. Therefore, the effects of a Put treatment on endogenous ethylene and the cell wall metabolism of blueberry fruit deserve further study.

**Figure 14:**
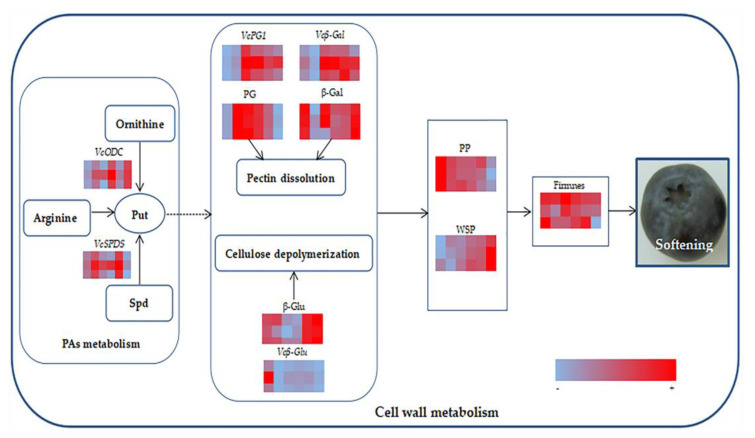
The potential model of exogenous Put treatment participating in cell wall metabolism and affecting blueberry fruit softening. Solid arrow presents direct regulation. Dotted arrow presents unclear regulation mechanism. Put: Putrescine. Spd: spermidine. PG: polygalacturonase. β−Gal: β−galactosylase. β−Glu: β−glucosidase. PP: protopectin. WSP: water−soluble pectin.

In summary, Put can delay the fruit softening process, which is related to the inhibition of the cell wall metabolic enzyme activity as shown in Figure 14. The results obtained are consistent with those of a study on carambola treated with Put [46]. The SSC was determined; organic matter in fruit can be converted into soluble sugar and other nutrients at the initial stage of the shelf life, so the SSC increased. However, in the later stage of storage, fruit respiration led to the continuous consumption of carbohydrates and organic matter, so the SSC decreased and was lower than that in the early stage of storage [47].

In order to explore the regulation of Put anabolism−related gene expression after the Put treatment, it can be seen in Figure 6 that the Put treatment promoted the expression of the *VcODC* gene and inhibited the expression of the *VcSPDS* gene. The decrease in the degree of fruit softening may also have been related to the regulation of endogenous Put anabolic gene expression by the exogenous Put treatment, affecting the cell wall metabolism of the fruit. Thus, the mechanisms of Put that increase the fruit firmness are not fully understood and require further verification. Based on the results of the transcriptome sequencing, the binding sites of the transcription factors were predicted, and the expressions of the key transcription factors were analyzed, which lays a foundation for subsequent experiments.

## 4. Material and Methods

### 4.1. Fruit Materials and Treatment

Blueberry fruits (*Vaccinium corymbosum* L., cv. Bluecrop) were harvested at Blueberry Base in Shenyang (41°39′55.82″ N, 123°05′12.46″ E), Liaoning Province. Blueberries of uniform size, color, and maturity (85–90%) and presenting no mechanical injury were selected. The fruits were precooled at 20 °C for 3 h. Six kilograms of fruit were divided randomly into two groups for the control and Put treatments. The control blueberries were soaked in distilled water for 10 min. The treated blueberries were soaked in 1 mM of Put for 10 min. After soaking, the blueberries were dried on filter paper and then packed into polyethylene terephthalate (PET) fresh−keeping boxes with 12 air holes. Each box contained 60 fruits and was wrapped with fresh-keeping bags (thickness of 0.2 mm) to prevent water loss. They were stored at 20 °C and 80% relative humidity. Samples were collected on days 0, 2, 4, 6, 8, and 10. The firmness, decay incidence, and SSC were measured on each sampling point. 100 g fruit was taken from each sampling point and frozen at −80 °C after quick−freezing them with liquid nitrogen. These fruits were used to extract RNA to measure the relative gene expression, cell wall substances, and cell wall metabolism enzyme activities. All measurements were independently performed in triplicate.

### 4.2. Decay Incidence

The apparent spot mildew and inedibility of blueberries in each box were regarded as the decay standard (An), and the decay incidence was expressed as a percentage of the total number of blueberries per box (Am). The decay incidence was determined at 0, 2, 4, 6, 8, and 10 d after harvest, and three independent replicates were measured. Decay incidence = An/Am × 100%.

### 4.3. Firmness

The fruit firmness was measured by a CT3 texture analyzer (Brookfield Engineering Laboratories, Inc., Middleboro, MA, USA) with a TA39 rod probe. The probe had a diameter of 2 mm and was used with a penetration depth of 7 mm and a test speed of 0.5 mm s^−1^. Two equatorial parts of 10 fruits were measured on each sampling point. The fruit with the peel was measured first, then the fruit was rotated 90° to remove the peel and measured again, for fruit firmness and sarcocarp firmness, respectively. The results are expressed in newtons (N).

### 4.4. SSC

A digital refractometer (PAL−13810, Otago, Tokyo, Japan) was used to determine the SSC. Each test used 3 blueberry fruits after grinding and filtering.

### 4.5. Pectin Content

One gram of frozen blueberries was crushed in liquid nitrogen on day 0, 2, 4, 6, 8, and 10. The WSP and PP were measured using the carbazole method, according to the method of Noussaire [48]. The fruit was extracted with 95% ethanol 4 times. Added 20 mL distilled water, kept in a 50 °C water bath for 30 min, and centrifuged (7104× *g*, 20 °C, 15 min). The supernatant was WSP determination solution. We added 25 mL of 0.5 mM sulfuric acid solution to the precipitation, boiled it for 1 h, and centrifuged (7104× *g* 20 °C, 15 min) again. The supernatant was PP determination solution. Taking 1 mL of each measuring solution respectively, we added 6 mL of concentrated sulfuric acid, boiled it in boiling water for 20 min, cooled it, and then added 0.2 mL of 1.5 g L^−1^ carbazole ethanol solution, keeping it away from light for 30 min. The absorbance was measured at 530 nm.

### 4.6. PG, β−Gal, and β−Glu Activity

Frozen blueberry fruits (10 g) were ground in an ice bath using 20 mL ethanol (95%), placed at a low temperature for 10 min, and then centrifuged (12,000× *g*, 4 °C, 20 min), discarded the supernatant. The process was repeated three times. Precooled extraction buffer (5 mL) was added to the precipitate, placed at 40 °C for 20 min, and then centrifuged (12,000× *g*, 4 °C, 20 min). The supernatant was crude enzyme solution, which was used to determine the activity of cell wall metabolic enzymes.

The PG activity was measured according to the method described by Ren [49], with modifications. The reaction mixture contained 1 mL of disodium hydrogen phosphate–citrate buffer (pH 4.0), 1 mL of pectin solution (1%), and 0.5 mL of crude enzyme extracts. The mixture was incubated at 37 °C for 1 h, and then the reaction was terminated by adding 1.5 mL of 3,5−dinitrosalicylic acid (DNS) reagent. Finally, it was heated with boiling water for 5 min. The absorbance was measured at 540 nm, and the pectin solution was not added to the control group. One enzyme activity unit was defined as the amount of galacturonic acid produced per hour per gram of sample fresh weight (FW) as μmol h^−1^g^−1^ FW.

The β−Gal activity was measured according to the method described by Zhang [50], with slight modifications. The reaction mixture contained 0.2 mL of crude enzyme solution and 2 mL of p−Nitrophenyl−β−d−Galactopyranoside (3 mM). The mixture was incubated at 37 °C for 1 h. Then, the reaction was terminated by adding 2 mL of Na_2_CO_3_ (1 M). The absorbance was measured at 400 nm. The boiling enzyme solution was used for the control. One enzyme activity unit was defined as the amount of p−nitrophenol produced per hour per gram of sample fresh weight as μmol h^−1^g^−1^ FW.

The β−Glu activity was measured according to the method described by Ji [51], with modifications. Salicin solution (1.5 mL, 10 g L^−1^) and 0.5 mL of crude enzyme solution were treated in a 37 °C water bath for 1 h. Then, 1.5 mL of DNS was used to terminate the reaction, and the absorbance was measured at 540 nm. One enzyme activity unit was defined as the amount of glucose produced per hour per gram of sample fresh weight as μmol h^−1^g^−1^ FW.

### 4.7. RNA Isolation and cDNA Synthesis

The total RNA was extracted from the blueberry fruit according to the OmniPlant RNA kit (CWBIO, Beijing, China). The samples were electrophoresed on 1.0% agarose gel, and the absorbance was measured at 260 nm to evaluate the quality of the total RNA. Reverse transcription was performed using a Hifiscript cDNA Synthesis Kit (CWBIO, Beijing, China), which was used as a template for real−time quantitative polymerase chain reaction (RT−qPCR) analysis and stored at −20 °C.

### 4.8. Gene Expression Analysis

RT−qPCR was used to analyze the relative gene expression with the UltraSTBR Mixture (Lox ROX) (CWBIO, Beijing, China). The gene expression level of the blueberry fruit on the day of harvest was set as 1, and the 2^−ΔΔCt^ method was used to calculate the expression [52]. The gene primers designed by Primer Premier 5.0 are shown in Table 1, according to Luo’s method [53]. The actin gene was used as an internal reference for the whole experiment. The reference species was *Vaccinium corymbosum* (Taxonomy ID: 69266). The transcriptome data refer to the study by Colle [54].

### 4.9. Cloning of the Full−Length Sequence of VcPG1 CDS

The PB.7565.1 gene sequence was obtained from the blueberry transcriptome. After a comparison with NCBI (https://www.ncbi.nlm.nih.gov/ (accessed on 19 September 2021)), primers for CDS were designed. The primer sequences are shown in Table 2 for PCR and sequencing. Gene cloning referred to Wu’s method [55].

### 4.10. Overexpression of VcPG1 in Blueberries

Full−length *VcPG1* cDNA was cloned into pRI101 plasmid (Takara Bio. https://www.takarabio.com/products/cloning/linkers-primers-and-cloning-vectors/cloning-vectors/plant-transformation-vectors/pri-101-dna-vectors (accessed on 31 March 2021)) to obtain the *VcPG1*−OE overexpression vector. Then, *Agrobacterium tumefaciens* strain GV3101 was transformed with *VcPG1*−OE. The Agrobacterium tumefaciens strain was inoculated into LB solid plates containing Kan and Rif and cultured for 3 d. A single colony was placed in a centrifuge tube, and 10 μL of ddH_2_O was added to the colony. The bacterial solution was added to LB liquid medium containing Kan and Rif and then cultured by oscillation at 28 °C for 28 h. After centrifugation (11,100× *g*, 20 °C, 2 min), the precipitate was suspended with an appropriate amount of ddH_2_O, and the OD_600_ was adjusted to about 1. We used a 50 μL microsyringe (marked to a 7 mm depth on the needle) to extract 20 μL of the Agrobacterium tumefaciens, which was vertically injected into the whole blueberry fruit from the fruit stem. The control fruit was injected with an empty vector. It was measured after 2 days.

### 4.11. Predictive Analysis of Cis−Acting Elements of Key Transcription Factors

The cis−acting elements of the key transcription factors were predicted using PlantTFDB (http://planttfdb.gao-lab.org/tf.php?sp=Ppe&did=Prupe.I004500.1.p (accessed on 10 July 2021)).

### 4.12. Statistical Analyses

All analyses were performed using SPSS20.0 software (BM Corp, Armonk, NY, USA). The data were analyzed by one−way analysis of variance (ANOVA) with Duncan’s multiple range tests. All figures were created using Origin 8.1 software (MicroCal Software Inc., Northampton, MA, USA).

## 5. Conclusions

In conclusion, the Put treatment had a positive effect on the quality of the fruit. Put maintained a high level of firmness by inhibiting the activity and expression levels of enzymes involved in cell wall metabolism, which may have been conducive to slowing the softening of the blueberries. *VcPG1* proved to be the key gene for fruit softening. The Put treatment regulated the expression of *VcODC* and *VcSPDS* genes, promoting endogenous mechanism changes. The binding sites of the key transcription factors were predicted and the expressions were analyzed. These results provide an experimental basis for blueberry fruit storage. In the next step, we plan to study the regulation of transcription factors on the *VcPG1* gene in blueberry and analyze the internal mechanism of blueberry softening from the molecular level.

## Figures and Tables

**Table 1:** Primers for RT−qPCR analysis.

	Forward Primer	Reverse Primer
Actin	5′−ACTACCATCCACTCTATCACCG−3′	5′−AACACCTTACCAACAGCCTTG−3′
*VcPG1*	5′−ACCACCAACCGCATTA−3′	5′−AAGCGTCAGGTGAGTAAG−3′
*VcPG2*	5′−ACGGTTCAGGGTGTCTGGAT−3′	5′−GGTTGGGTGGTGTGTTTGCT−3′
*VcPG3*	5′−CAGGGTCATGTGGCTGGTA−3′	5′−AGACGGGCGGACGCTTAA−3′
*Vcβ−Gal*	5′−CTTCTCTCTCTTCTCGCCGC−3′	5′−CGAATGCCTTTGCCCTCAAC−3′
*Vcβ−Glu*	5′−TCGACCGAAGCGTCGCTACT−3′	5′−GTCCACCATGTCCGCCCAAT−3′
*VcSPDS*	5′−TTCGTCGTCTCCACCTCATC−3′	5′−CCAGTTTCCTTGCCCAATTC−3′
*VcODC*	5′−GCGAACCCTAACAGCCACCA−3′	5′−TACCACGCCCAAGTCCAGCA−3′
VcNAC26	5′−GATGGCATCCGGTGCTTCTCC−3′	5′−GAAGTGGACGACAAGCTCCTCATC−3′
VcNAC23	5′−TTCATTGGTGGTTCCGCGAGAAG−3′	5′−GTCGATGGAAGGAACATCCTGCTC−3′
VcNAC33	5′−GCGAACGAACAGATTGGGTAATGC−3′	5′−CCAGGTCCGCTCTTCTTGAACAC−3′
VcNAC35	5′−CCAGTGTTCACAAGTGATGGCAAG−3′	5′−GGAGCCTGAACTCCTGCATTATCC−3′
VcNAC43	5′−TCCAGAGCGAGCCGACGAAG−3′	5′−GCAACGGCGAGGTGAAGGTG−3′
VcNAC46	5′−TCCAGAGCGAGCCGACGAAG−3′	5′−CGAGGTGAAGGTGCTGTCCATG−3′
VcNAC62	5′−TGCTTCTCCAGCCACAGT−3′	5′−ACAACCGATTGACCTCCT−3′
VcbZIP2	5′−ATGGCGATGGCAATGGGAAA−3′	5′−AGGGCTCGAAGCCGAGAGAA−3′
VcMYC2	5′−GTAAAGGACCACCCCCACCT−3′	5′−CGTTCACGACCGACACACTC−3′
VcWRKY1	5′−AGAAAAGAGGATGGGGAGGA−3′	5′−ATGATGATGATGGTGGTGGC−3′
VcMYB1	5′−CTGCTCATCCTTACCCACA−3′	5′−TTCCACCGCCATCAATAC−3′
VcPHL7	5′−AGGGATACTGCTCCAACT−3′	5′−GCAAGGCGATACTTCTGT−3′

**Table 2:** The primer sequences for the *VcPG1* CDS.

	Forward Primer	Reverse Primer
VcPG1	5′−ATCCTCCTCCCCATCCTCCT−3′	5′−TACAAATCATTACAAGTTTATCTAAGCACA−3′

## Data Availability

Data are available from the authors upon request.
